# Needs and Preferences Among Food Pantry Clients

**DOI:** 10.5888/pcd18.200531

**Published:** 2021-04-01

**Authors:** Caitlin E. Caspi, Cynthia Davey, Christina Bliss Barsness, Nora Gordon, Laura Bohen, Marna Canterbury, Hikaru Peterson, Rebekah Pratt

**Affiliations:** 1 Rudd Center for Food Policy and Obesity, University of Connecticut, Hartford, Connecticut; 2 Department of Allied Health Sciences, University of Connecticut, Storrs, Connecticut; 3 Program in Health Disparities Research, Department of Family Medicine and Community Health, University of Minnesota, Minneapolis, Minnesota; 4 Biostatistical Design and Analysis Center, Clinical and Translational Science Institute, University of Minnesota, Minneapolis, Minnesota; 5 University of Minnesota Extension, St Paul, Minnesota; 6 HealthPartners, Bloomington, Minnesota; 7 Department of Applied Economics, University of Minnesota, St Paul, Minnesota

## Abstract

**Introduction:**

Food pantries serve households in need, including many with a family member with a diet-related chronic disease, yet data on client priorities to inform hunger relief practices are lacking. We used a statewide client survey in Minnesota to determine needs and priorities of food pantry clients in 2017 and 2019 and to identify how well Minnesota pantries met those needs in 2019.

**Methods:**

Our survey was administered in 2017 and 2019. Food pantries in Minnesota were mailed 25 surveys each, with instructions for administering the surveys anonymously to clients. Descriptive analyses compared 2017 and 2019 data and compared client priorities for foods and services with how often they were available at the pantry in 2019.

**Results:**

The 2017 survey represented 4,321 clients from 188 pantries; the 2019 survey represented 5,529 clients from 220 pantries. Most measures of food pantry use were consistently high across the years; about three-quarters of clients had been visiting the pantry for a year or more. In 2019, 85% of clients said it was important to have fresh fruits and vegetables, but only 52% said these were always available. About two-thirds had a household member with a diet-related chronic disease. The ability to choose their own foods was clients’ top priority.

**Conclusion:**

The types of food most requested by clients tended to be healthy but were inconsistently available. Most important to clients was being able to choose their own food. Results underscore the need for continued monitoring of client priorities.

**Summary**

**What is already known on this topic?**

Large-scale surveillance data on the needs of food pantry clients are almost nonexistent.

**What is added by this report?**

This report presents data from 2 years of a Minnesota statewide client survey from 2017 (n = 4,321) and 2019 (n = 5,529), designed and executed by a coalition of antihunger practitioners and researchers.

**What are the implications for public health practice?**

Procuring food to meet client demand requires coordinated efforts at multiple levels of the hunger relief system. Results suggest the importance of continued monitoring of client needs and priorities.

## Introduction

Hunger relief agencies such as food pantries serve people with a disproportionate burden of diet-related health conditions (1–4). However, this setting has until recently been a neglected area of research, cross-sector collaboration, and public health promotion. Clients using food pantries, especially those with chronic disease, often want healthy foods (5–9), a need that does not always match pantry staff perceptions of client needs (10–13). Small-scale and localized research studies have also suggested that clients rely on food pantries for large portions of their food (14,15) over long periods of time (1,14,16). The persistent nature of food insecurity and the chronic food needs of clients have been identified by hunger relief agencies in their recent move away from the term “emergency food system,” prompting food pantries to adopt new policies and interventions to improve the healthfulness and client-centeredness of their services (4,14,17–21).

Despite a broad, emerging understanding of chronic client food needs, food pantries are a historically fragmented and resource-strained system (4), making it difficult to obtain data and monitor system needs and capacity. Large-scale data on characteristics of pantry clients are rare (1). Surveillance data capturing changes in pantry environments and client experiences are almost nonexistent. These data are necessary to inform policy, practice, and programming of hunger relief organizations and to establish the next stages of systems change.

Our study used data from a food pantry client surveillance survey administered in Minnesota, from November 2017 through February 2018 and from November 2019 through February 2020. Our objective was to describe food pantry client needs and priorities in 2017 and 2019, identify client priorities for foods and services received at the pantry, and assess how well Minnesota pantries met those priority needs in 2019.

## Methods

The 2017 and 2019 Minnesota Statewide Food Pantry Client Surveys were designed and implemented as a collaborative effort among hunger relief agencies statewide. Hunger relief agencies that directly distribute food at no cost for home consumption are known as food pantries in most states, and we refer to them as such in this article. However, these agencies are called food shelves in Minnesota and were referred to as such in the Minnesota surveys. The surveys were conceived and developed by a team of multisector partners, which included the University of Minnesota, and were developed to provide data to local pantries on their clients’ needs. The 2017 survey contributed formative data for intervention development for a study funded by the National Institutes of Health that is ongoing in 16 pantries.

### Data collection

In the fall of 2017, a 16-item survey was mailed to 403 food pantries across the state. The list was obtained from Hunger Solutions Minnesota, an antihunger policy and advocacy agency that administers The Emergency Food Assistance Program (TEFAP) (22) and provides support to pantries throughout Minnesota. Each mailed packet contained 25 blank surveys, 25 sealable envelopes, and a large prepaid mailing envelope for pantries to return the surveys. The 2017 surveys were available only in English.

Pantries were provided instructions on how to give the self-administered survey to 25 client volunteers whose responses would remain anonymous and confidential. To encourage participation by a large number of pantries, survey administration processes were designed to minimize pantry burden; therefore, documenting the refusal rate was not required. For participating, pantries received an infographic report of their pantry-specific results along with a 2-page summary of the statewide results (https://www.supershelfmn.org/resources). Pantries were also entered into a random drawing for a $200 food credit to their food bank. This study was approved by the University of Minnesota Institutional Review Board (STUDY00009859).

Similar procedures were followed in 2019, with some differences. An additional 13 questions were added to the 2017 survey, and the 2019 survey was offered in 3 languages besides English: Spanish, Somali, and Hmong. Clients who agreed to participate received an information sheet about the survey. The survey was mailed to the 367 food pantries receiving TEFAP according to the list provided by Hunger Solutions Minnesota and was also accessible through a website for download. Pantries participating in the 2019 survey were entered into a random drawing to receive a $400 check.

### Measures

In both years, survey items included questions around 1) categories of food clients most wanted at their visit (from a list of 18 food categories), 2) food pantry use, 3) amount of food obtained at the food pantry, 4) food insecurity, 5) other food assistance used, 6) chronic disease, and 7) demographics.

To assess foods categories clients most wanted at their food pantry, the survey asked “Which of these foods are important to you to have every time you visit the food pantry?” Clients could select all that applied. In 2017, 18 response options ranged from fresh fruits and vegetables to candy. The 2019 survey also included plain, nonwhole grains, nondairy products, and culture-specific foods. Response options were open-ended to include culture-specific foods and other foods.

To determine food pantry use, clients were asked “About how often do you visit this food pantry?” Response options were “once a week or more,” “a few times a month,” “once a month,” “once every other month,” “a few times a year,” “once a year or less often,” and “this is my first time visiting this food pantry.” Clients were also asked “About how long have you been visiting this food pantry?” Response options were “this is my first time,” “about a month,” “about 6 months,” “about a year,” and “more than a year.”

To assess the amount of food obtained at the pantry, clients were asked “In the last 6 months, how much of all the food you got was from this food pantry?” and “In the last six months, how much of all your fruits and vegetables was from this food pantry?” Responses were” I didn’t get any,” “less than half,” “about half,” “more than half,” or “all of my food.”

A validated 2-item question (23) was used to determine food insecurity of respondents. Responding yes to either or both of these 2 items indicated a food insecure status.

To assess other food assistance use, participants were asked whether, in the last 12 months, they used other food assistance resources such as SNAP (Supplemental Nutrition Assistant Program), WIC (Special Supplemental Nutrition Program for Women, Infants, and Children), school meals (free and reduced-price lunch, free breakfast), or another food pantry. To assess chronic disease, participants were asked “Has a doctor or health care professional ever said that you or someone in your household should lose weight, has high blood pressure, has high cholesterol, has heart disease, or has diabetes (or high blood sugar, including borderline or prediabetes)?”

Demographic questions were race, household size, number of children (0–18), number of seniors (aged ≥60 in 2017 and ≥65 in 2019), and gender as an open-ended item in 2019.

New survey questions in 2019 included household expense tradeoffs (“In the past year, have you had to choose between buying food and [paying for] utilities, transportation, housing, medical care/medicine, education?”) with response options yes or no; a household food adequacy question (“Do you get enough food to meet your household needs?”) with response options yes or no; and availability of the 5 most important food categories from the 2017 survey (available always, often, sometimes, rarely, or never). Clients were also asked, “When considering your experience at the food pantry, what 3 things are most important to you?” with instructions to pick the top 3 of 11 items (eg, wait time is reasonable), and “How often do you experience the following at the food pantry?” with response options of always, sometimes, never. Questions in 2019 had an option of “prefer not to answer.” All data were entered into REDCap (REDCap Consortium). Data quality assurance included performing quality checks for REDCap field errors and selecting a sample of 5% of surveys to verify accuracy of data entry between the paper survey and REDCap versions.

In 2017, responses were received from 188 pantries (response rate 46.7%) and 4,321 clients. In 2019, 221 food pantries (response rate 57.5%) and 5,559 clients responded. To focus results on Minnesota food pantries, the 28 surveys from the sole participating pantry in Wisconsin were excluded; 2 surveys missing more than half of their data were also excluded. The 2019 analysis sample had 220 pantries and 5,529 clients. Because of the data collection method, we do not know the proportion of clients who participated in both the 2017 and 2019 surveys. Four pantries in 2017 and 17 pantries in 2019 returned responses without indicating the name of their pantry and, therefore, missing geographic data.

### Analysis

Descriptive statistics included frequencies and percentages for categorical survey items (calculated from nonmissing data in 2017 and non-“prefer not to answer” responses in 2019) and mean (SD) for continuous items. Frequencies and percentages of urban and rural clients were calculated on the basis of Rural–Urban Commuting Area (RUCA) code designation (codes 1–3 for urban, codes 4–10 for rural) (24) of the food pantry.

Bar charts of the top 5 foods important for clients to have at each visit were created, representing the percentage of clients who 1) selected the food as important to have at each visit, with no limit on the number of food categories clients could select, and 2) reported that the food category was always available at their pantry visit.

Client responses to the 11 food pantry experiences were aggregated to the pantry level. Boxplots were created to represent the pantry-level distributions of the percentage of clients 1) selecting each experience as one of top 3 most important and 2) indicating that each experience was always experienced. Boxplot features included a box spanning the interquartile range (25th–75th percentile), a line at the median value, a marker at the mean, “whiskers” that extend to the maximum and minimum values within 1.5 interquartile range above the 75th percentile and below the 25th percentiles, and markers for aggregated percentages beyond the whiskers.

## Results

In 2017, 46.6% of the sample came from urban food pantries, and 53.4% came from rural pantries (Table 1). Households averaged 3.2 people; half (50.6%) included children and just over one-third (37.6%) included seniors. Household members were mostly White (69.2%), with 6.9% Black or African American, and 20.1% identifying as other racial categories; 7.5% of family members identified as Hispanic. Sample demographic characteristics and geography were similar in 2019, except for a higher percentage of clients from urban food pantries (52.5%) and a slightly lower percentage of households with any children (44.9%). The percentage of households reporting any seniors in the household was also lower, but this could be due to the different definitions of senior in the 2 surveys (age ≥60 in 2017 and ≥65 in 2019).

**Table 1:** Characteristics and Geography of Food Pantry Clients in Minnesota, Statewide Surveys, 2017 and 2019

Characteristics^a^	2017 Clients, N = 4,321^b^	2019 Clients, N = 5,529^c^
**Food pantry location**		
Urban (RUCA codes 1–3)	1,989 (46.6)	2,771 (52.5)
Rural (RUCA codes 4–10)	2,278 (53.4)	2,507 (47.5)
**Race**		
American Indian or Alaska Native	204 (4.8)	266 (5.1)
Asian	45 (1.1)	75 (1.4)
Black or African American	290 (6.9)	475 (9.1)
Native Hawaiian or Other Pacific Islander	11 (0.3)	11 (0.2)
White	2,917 (69.2)	3,449 (66.2)
Other (including more than one race or prefer not to answer)	747 (17.7)	937 (18.0)
**Ethnicity**		
Hispanic	314 (7.5)	377 (7.2)
Non-Hispanic	3,900 (92.5)	4,836 (92.8)
**Survey language^d^**		
English	4,321 (100)	5,455 (98.7)
Spanish	NA	65 (1.2)
Somali	NA	8 (0.1)
Hmong	NA	1 (0.02)
**Urban client distance traveled to food pantry, miles**		
<1	490 (25.0)	651 (23.8)
≥1	1,471 (75.0)	2,078 (76.2)
**Rural client distance traveled to food pantry, miles**		
<5	1,308 (59.8)	1,442 (58.0)
≥5	878 (40.2)	1,045 (42.0)
**Household composition^e^**		
Any children (<18 y) in household	2,115 (50.6)	2,170 (44.9)
Any seniors (2017: ≥60; 2019: ≥65) in household	1,555 (37.6)	1,574 (33.2)
**Number of people in household, mean (SD)**	3.2 (2.0)	3.0 (2.0)

Abbreviations: NA, not applicable; RUCA, Rural-Urban Commuting Area.

a Values are number (percentage) unless otherwise indicated.

b Percentages reported are of nonmissing data. Data are for 188 food pantries. RUCA codes were unavailable for 4 pantries because of unknown addresses.

c Percentage of nonmissing and non–prefer-not-to answer (except for race/ethnicity) responses in 2019. Data are for 220 food pantries. RUCA codes were unavailable for 17 pantries because of unknown addresses.

d The survey was offered only in English in 2017.

e Household composition rows do not add up to 100% because categories are not mutually exclusive or exhaustive (ie, households may contain both children and seniors, or contain neither children nor seniors).

By numerous metrics, participants relied consistently on the food pantry as a substantial source of household food. In 2017, about three-quarters of participants (74.4%) had been visiting their food pantry for about a year or more (Table 2). More than three-quarters (76.9%) visited the food pantry about once a month or more. Just over half (53.4%) reported that in the past 6 months they received half or more of their total food from the food pantry, and 42.6% received half or more of all fruits and vegetables from the pantry in the past 6 months. Participants also received support from numerous other food assistance programs. Nevertheless, 67.3% reported food insecurity. Reliance on these food resources was similar in 2017 and 2019, with 2 exceptions. In 2019, 59.6% of participants reported getting half or more of their total fruits and vegetables from the food pantry, 17 percentage points higher than in 2017. In addition, in 2019, 49.8% of client reported that they used SNAP in the last 12 months, 7.3 percentage points higher than in 2017. In 2019 only, 71.9% of participants said that they got enough food to cover their household needs.

**Table 2:** Food Resources and Food-Related Needs Among Clients Visiting Food Pantries in Minnesota, Statewide Surveys, 2017 and 2019

Survey Item	2017, N = 4,321^a^, n (%)	2019, N = 5,529^b^, n (%)
**Visited pantry for about a year or more**	3,186 (74.4)	3,973 (73.4)
**Visits pantry about once a month or more**	3,284 (76.9)	4,135 (75.6)
**About half or more of all food was from pantry in past 6 months**	1,971 (53.4)	2,478 (55.4)
**About half or more of all fruits and vegetables was from pantry in past 6 months**	1,594 (42.6)	2,670 (59.6)
**Food insecure^c^**	2,843 (67.3)	3,668 (69.0)
**Would like to provide more fruits and vegetables for family**	3,973 (93.0)	4,733 (92.5)
**Someone in household knows how to prepare many fruits and vegetables**	3,775 (89.6)	5,054 (96.3)
**Received or used in the last 12 months**		
SNAP	1,836 (42.5)	2,509 (49.8)
School meals	574 (13.3)	834 (15.1)
Other food pantry	514 (11.9)	640 (11.6)
WIC	471 (10.9)	526 (9.5)
**A doctor told respondent or a household member that they**		
Should lose weight	1,562 (37.5)	1,872 (40.6)
Have hypertension	1,800 (43.2)	2,208 (46.7)
Have high cholesterol	1,328 (32.0)	1,529 (33.4)
Have diabetes or prediabetes	1,177 (28.3)	1,469 (31.9)
Have heart disease	619 (14.9)	757 (17.0)
**Someone in household has any of the above conditions**	2,800 (66.8)	3,376 (67.7)
**Do you get enough food to cover household needs^d^**	NA	3,489 (71.9)
**In the past year, have you had to choose between buying food and paying for:^d^**		
Utilities	NA	2,299 (41.6)
Transportation	NA	1,621 (29.3)
Housing	NA	1,553 (28.1)
Medical care / medicine	NA	1,035 (18.7)
Education	NA	201 (3.6)

Abbreviations: NA, not applicable; SNAP, Supplemental Nutrition Assistance Program; WIC, Special Supplemental Nutrition Program for Women, Infants, and Children.

a Percentages reported are nonmissing data.

b Percentages reported are nonmissing data and non–prefer-not-to-answer responses.

c Answered yes to either or both of 2 questions about the past 12 months: “We worried about whether our food would run out before we got money to buy more,” and “The food we bought just didn’t last and we didn’t have money to buy more.”

d Included in 2019 survey only.

In 2017, most participants (66.8%) reported that they or a household member had been told that they had 1 or more of 5 chronic health conditions (should lose weight, had hypertension, had high cholesterol, had diabetes or prediabetes, or had heart disease). This percentage was similar in 2019 (67.7%). Almost all (93.0%) participants wanted more fruits and vegetables for their family in both 2017 and 2019. Most reported in 2017 (89.6%) and 2019 (96.3%) that someone in their household knew how to prepare many fruits and vegetables. Many participants in 2019 reported making tradeoffs in their household between buying food and paying for utilities (41.6%), transportation (29.3%), housing (28.1%), medical care or medicine (18.7%), and education (3.6%).

**Figure 1:**
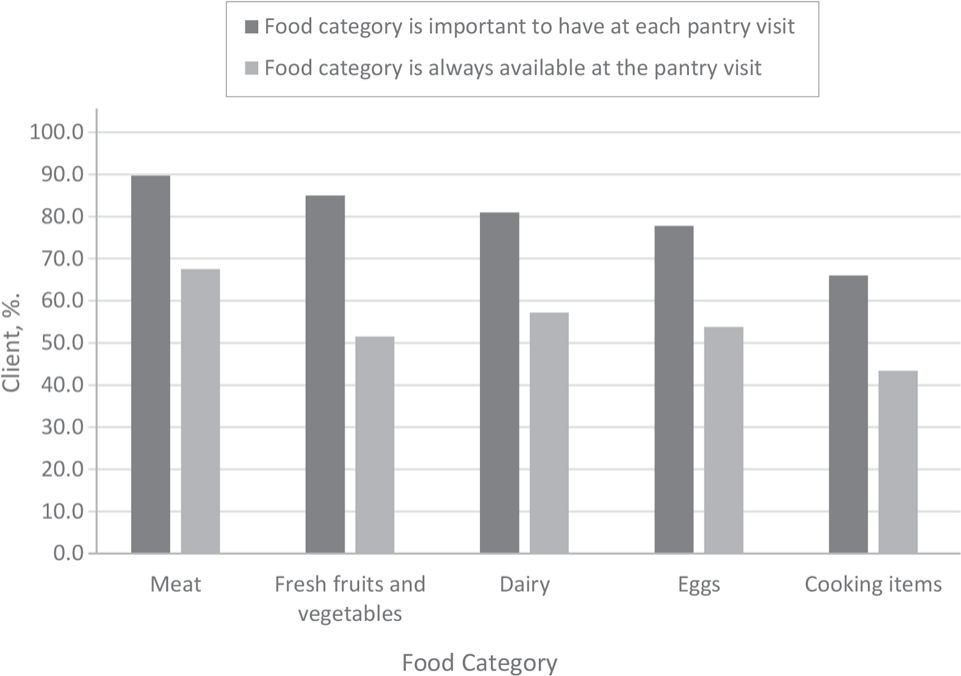
Priority food categories for food pantry clients in 2019 and whether those food categories were always available. The percentage of clients who reported each food category as a priority each time they visited the pantry and the percentage who reported that the food category was always available are also shown. Data are from 5,529 clients and 220 food pantries who participated in the 2019 Minnesota Statewide Client Survey (25).

In 2017, the top 5 most commonly marked types of foods that participants indicated were important to have every time they visit the food pantry were meat, poultry, and fish (91.2%); dairy (83.6%); fresh fruits and vegetables (82.8%); eggs (79.7%); and cooking items (eg, spices, oil) (62.3%) (Table 3). Client preferences stayed relatively stable in 2019, except that fresh fruits and vegetables moved up to the second most important item (Figure 1). Candy and soda were among the least important foods in both years; in 2019, culture-specific food was the least commonly selected food category to have available. In 2019, meat was reported by 67.5% of food pantry clients as being always available, whereas only 43.4% reported that cooking items were always available. Just over half of clients reported that fresh fruits and vegetables, dairy, and eggs were always available.

**Table 3:** Priority Foods for Each Visit Among Clients Visiting Food Pantries in Minnesota, Statewide Surveys, 2017 and 2019^a^

Food	2017, N = 4,321	2019, N = 5,529
Meat, poultry, fish	3,942 (91.2)	4,960 (89.7)
Dairy	3,611 (83.6)	4,480 (81.0)
Fresh fruits and vegetables	3,576 (82.8)	4,700 (85.0)
Eggs	3,444 (79.7)	4,304 (77.8)
Cooking items (eg, spices, oil)	2,690 (62.3)	3,649 (66.0)
Soup	2,593 (60.0)	3,255 (58.9)
Canned fruits and vegetables	2,465 (57.1)	3,082 (55.7)
White bread (sliced, hot dog buns, hamburger buns)	2,378 (55.0)	3,024 (54.7)
Peanut butter/Nut butters	2,366 (54.8)	3,193 (57.8)
Canned or boxed meals (ravioli, Hamburger Helper, mac and cheese)	2,306 (53.4)	2,594 (46.9)
Whole grains	2,106 (48.7)	2,693 (48.7)
Pastries (donuts, cakes, cookies)	1,594 (36.9)	2,350 (42.5)
Dried and canned beans	1,519 (35.2)	1,952 (35.3)
Nuts	1,285 (29.7)	2,046 (37.0)
Chips	1,205 (27.9)	1,766 (31.9)
Dried fruits and vegetables	1,102 (25.5)	1,649 (29.8)
Soda	860 (19.9)	1,211 (21.9)
Candy	749 (17.3)	1,034 (18.7)
Plain non-whole grains (white flour tortillas, non-whole grain pasta, white rice)^b^	NA	1,989 (36.0)
Nondairy products (eg, nondairy milk, nondairy cheese or yogurt)^b^	NA	1,384 (25.0)
Culture-specific foods^b^	NA	347 (6.3)

Abbreviation: NA, not applicable.

a Values are number (percentage). Number is number of clients who checked each food item. Percentages reported are of total number of client surveys in each year.

b Not included in the 2017 survey.

**Table:** 

Food Type	Clients Reporting Food Category as a Priority for Each Pantry Visit, %	Clients Reporting That Food Category Was Always Available at Pantry Visit, %
Meat	89.7	67.5
Fresh fruits and vegetables	85.0	51.5
Dairy	81.0	57.2
Eggs	77.8	53.8
Cooking items	66.0	43.4

**Figure 2:**
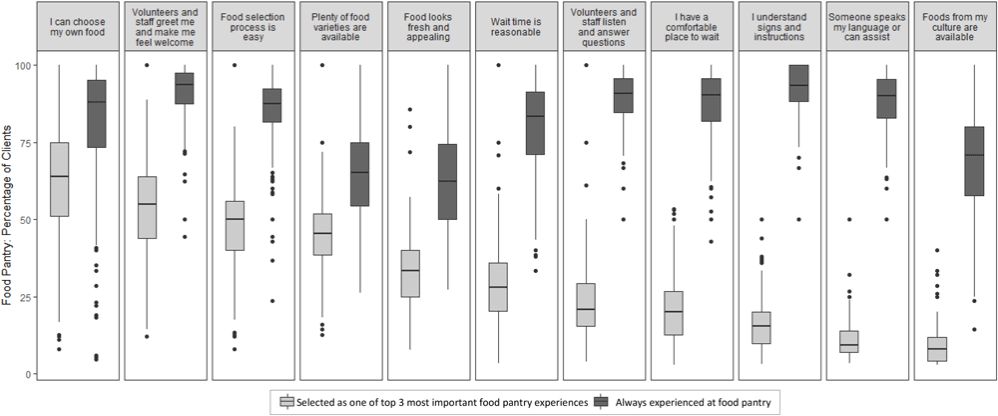
Most important experiences at the food pantry for clients, and whether clients always experienced them. Different experiences at the food pantry visit are listed on the x axis. The aggregated percentage of food pantry clients who selected each experience as one of the top 3 most important is presented. Also presented is the aggregated percentage of clients reporting that they always had that experience at the pantry. Data are from 5,529 clients and 220 food pantries participating in the 2019 Minnesota Statewide Client Survey (25).

Most important to clients was being able to choose their own food, being greeted and made to feel welcome by volunteers and staff, and having an easy process of food selection (Figure 2). Most clients who selected these experiences as important also reported that they always experienced them at the pantry. However, “always” responses varied considerably across pantries for many of the items.

## Discussion

Results from the 2 survey waves highlight several key findings about the food needs and preferences of clients. First, client reliance on food pantries was high and mostly consistent across the years: most clients reported visiting pantries once a month or more for a duration of about a year or more and getting about half or more of their total food from the pantry. These data suggest that food pantries contribute a substantial amount of food to client diets, and changes in food pantry food supply could have measurable effects on client diet quality and food security. Second, from the client perspective, the types of food most important to have at the pantry were healthy, staple foods. Yet these foods were inconsistently available to clients at their food pantry visit. Third, the overwhelming majority of clients reported that they would like to provide their household with more fruits and vegetables and that someone in their household knew how to prepare many fruits and vegetables. These statewide results are largely consistent with smaller-scale studies from different geographic regions that revealed a strong demand for healthy foods despite a common perception that clients preferred convenience foods (5–13). Taken together, results suggest that efforts are needed to procure more healthy, staple foods to meet client demand. Acquiring these foods requires coordinated efforts at all levels of the hunger relief system. Results on client demand are relevant to agencies involved in TEFAP food procurement and distribution and to food banks, which are a critical food stream for pantries.

At the food pantry level, procurement changes likely require budgetary tradeoffs, because priority foods tend to be more expensive than other types of nonperishable foods (26). Although challenging, a multipronged approach at the food pantry for meeting client demand in the top 5 categories might involve 1) creating new food procurement streams, such as rescuing healthy, perishable food from retail food stores; 2) spending fewer organizational dollars on lower-demand types of food that may, instead, be donated in abundance, such as boxed meals and processed snacks; 3) rebudgeting to allow increased spending on more expensive items, such as cooking oil and spices, that allow people to cook meals at home; 4) soliciting donations (or cash to be used) for specific high-demand or harder-to-source items to fill in needed gaps in procurement. Given that most of clients reported that someone in their household knew how to prepare many types of fruits and vegetables, budgeting tradeoffs might also carefully consider the type and scope of investments in nutrition education programming.

Other survey results draw attention to less frequently discussed aspects of the food pantry client experience. Clients valued having experiences at the pantry that are likely to be considered cornerstones of good customer service in any service industry: welcoming staff, an easy process, and reasonable wait times. Being able to choose their own food at the food pantry was the top priority; other food-related features that clients prioritized were having plenty of varieties of food and having the food look fresh and appealing. Responses ranged widely across pantries in the degree to which many of these practices were implemented, signaling the need for greater consistency of client-centered practices. These results are consistent with smaller-scale studies citing the importance of being treated with dignity for clients visiting pantries (27,28).

Clients receiving food from the pantry faced complex health, financial, and social needs. Clients relied on a network of other food assistance programs beyond the pantry, often needed to make tradeoffs between food and other essentials, and had a high prevalence of reported chronic disease in their households. Taken together, these needs suggest that a wide social and health care safety net is essential for clients. Understanding these needs is essential for pantries, both to provide clients with the amount and types of food they need and to connect them with a broader safety net through wraparound services or referrals, where possible.

In general, responses in 2017 and 2019 were similar. However, significance testing was not performed to formally test time trends, and differences in the sample of participating pantries and clients between the 2 surveys (eg, location of pantries, race/ethnicity of participants) could account for the differences. Notably, data from our study were collected just before the novel coronavirus (COVID-19) pandemic. Although comprehensive recent data on the pandemic’s effect on hunger relief reliance are lacking, early evidence suggests that food insecurity rose substantially at the beginning of the pandemic (29), prompting an exceptionally high number of visits to food pantries, including a population of clients who were new to the system (30). Trends from the last recession a decade ago suggest that food insecurity rates and increased pantry reliance could remain elevated for an extended period of time (31). Moreover, because of the COVID-19 pandemic, new food distribution processes often meant less choice for clients, because food pantries tried to serve more people with minimal contact (32). Unclear is whether or how quickly food distribution practices surrounding client choice will return to prepandemic levels. Ongoing data collection in the hunger relief setting is necessary to monitor any changes in client food-related needs in this new era.

Our study has limitations. It presents data from clients in a single state; thus, results may not be generalizable to other geographic regions. To gather data in this low-resource setting, responses were collected from a convenience sample of clients, and the refusal rate is unknown. Possibly, clients who declined to participate were different from those who participated. Additionally, not all pantries in the state participated, and the pantries that did possibly represented higher-resourced pantries. Finally, sampling differences between the 2 surveys made it difficult to draw direct comparisons of prevalence across the 2 groups over time. In the future, analyses using more complex sampling and analytic techniques would allow for an interpretation of time trends, which would be particularly useful to conduct pre and post pandemic. Nevertheless, the sample is large and provides greater detail about the food pantry client experiences than is typically available in the hunger relief system.

Clients rely on food pantries for long periods of time and for a substantial portion of their household foods. The types of food most requested by clients tend to be healthy but are inconsistently available. Clients value customer service and choice in the pantry; they also face complex health and social needs. Results of our study underscore the need for continued monitoring of client food-related needs, particularly in light of current food insecurity trends and the potential for new patterns of use in the pandemic and post-pandemic era.
